# Emerging Robotic Innovations and Artificial Intelligence in Endotracheal Intubation and Airway Management: Current State of the Art

**DOI:** 10.7759/cureus.42625

**Published:** 2023-07-28

**Authors:** Muhammad Jaffar Khan, Arunabha Karmakar

**Affiliations:** 1 Anesthesiology and Perioperative Medicine, Hamad Medical Corporation, Doha, QAT

**Keywords:** airway management, simulations, artificial intelligence, robotic endotracheal intubation, artificial intelligence and robotics in healthcare

## Abstract

Robotic sciences have rapidly advanced and revolutionized various aspects of medicine, including the field of airway management. Robotic endotracheal intubation is an innovative method that utilizes robotic systems to aid in the accurate placement of an endotracheal tube within the trachea. This cutting-edge technique shows great promise in improving procedural precision and ensuring patient safety. In this comprehensive overview, we delve into the present status of robotic-assisted endotracheal intubation, examining its advantages, obstacles, and the potential implications it holds for the future. In addition, this review encompasses a comprehensive analysis of the existing literature and references on recent advances in robotic technology and artificial intelligence related to airway management.

## Introduction and background

Airway management forms a critical domain in anesthesia, resuscitation, and critical care medicine. Failure to successfully perform tracheal intubation or the misplacement of the tracheal tube can have life-threatening consequences, including asphyxia, hypoxemia, hypoxic cardiac arrest, and pulmonary aspiration. These complications can lead to severe morbidity and mortality. Clearly, it has been a focal point of extensive research and innovation, aiming to improve procedural efficiency and patient safety. The 4th National Audit Project (NAP4) conducted by the Royal College of Anaesthetists and Difficult Airway Society is recognized as the world’s most extensive audit of airway complications. NAP4 described significant airway management-related complications such as death, brain damage, emergency surgical airway procedures, or unexpected admission to the intensive care unit in various settings over a year throughout the United Kingdom. The incidence of major airway complications during general anesthesia was 46.3 per million cases (95% confidence interval (CI) = 38.4-54.2). Notably, these incidents were more frequent and severe in emergency and intensive care departments [1-3]. To mitigate the adverse outcomes associated with difficult airways, guidelines were developed for airway procedures. These guidelines are updated to incorporate advancements in medical knowledge and technology.

The fields of artificial intelligence (AI) and machine learning are expanding rapidly and have started to play strong roles in difficult airway management. However, new technologies necessitate thorough investigation to ensure their use leads to positive and measurable patient-centered outcomes, while avoiding any potential adverse effects [4-6].

Understanding the advancements and outcomes associated with robotic endotracheal intubation is crucial for healthcare professionals, researchers, and policymakers to make informed decisions regarding its integration into clinical practice. In this review, we aim to thoroughly examine and analyze the current status of robotic endotracheal intubation, offering a comprehensive understanding of the subject matter. By examining existing literature, we explore the benefits, challenges, and future implications of this innovative technique.

## Review

Methods

A literature search was conducted on PubMed and Google Scholar using [‘robotics’, ‘artificial intelligence’ or ‘technology’] and [‘endotracheal intubation’, or ‘airway management’] without date limitations. The search included review articles, clinical trials, editorials, animal studies, and observational studies without date limitations. After selecting articles from databases, a manual search was conducted to find additional relevant articles. Inclusion and exclusion of the articles were based on the relevance to the use of robotics or AI in endotracheal intubation or airway management.

Revolutionizing airway management: the role of mechanical robots

Robotic endotracheal intubation is a novel technique that combines the use of automated systems with the expertise of healthcare professionals to secure the airway during various medical procedures. This harnesses the precision, control, and visualization capabilities of robotic systems. Biomedical engineers and healthcare professionals have developed various types of robots to achieve successful and accurate endotracheal intubation. These include robotic assistance systems with specialized instruments and cameras, teleoperated robots controlled remotely, and automated robots utilizing AI and advanced algorithms to automate the entire process of endotracheal intubation. While these systems show promise, they are still being refined and evaluated for integration into clinical practice (Table 1).

**Table 1:** Advantages and disadvantages of the current robotic intubation system.

Advantages	Disadvantages
Enhanced procedural precision	High cost and limited availability
Improved visualization	Need for specialized training and expertise
High-quality patient care	Technical difficulties during the procedure
Improved ergonomics	Delays due to machine setup
Remote guidance by experienced personnel	Bulky and transport challenges

DaVinci robotic system for endotracheal intubation

Extensive research has been conducted over the past decade to investigate the involvement of robots in the realm of airway management. Tighe et al. in 2010 described the first documented case of a simulated robotic-assisted fibreoptic intubation using the versatile DaVinci Surgical System Type S (DVS) and airway simulation mannequin [7]. The DVS system consisted of four robotic arms, with one arm equipped with a high-definition stereoscopic camera. The workstation enabled the operator to observe the camera feed, manipulate the robotic limbs, and simultaneously receive video input from external sources. Successful fibreoptic intubation was achieved using both the oral and nasal approaches. It took 75 seconds for the bronchoscope to reach the carina from the oropharynx during oral intubation, while nasal intubation took 67 seconds.

The Kepler Intubation System

**Figure 1:**
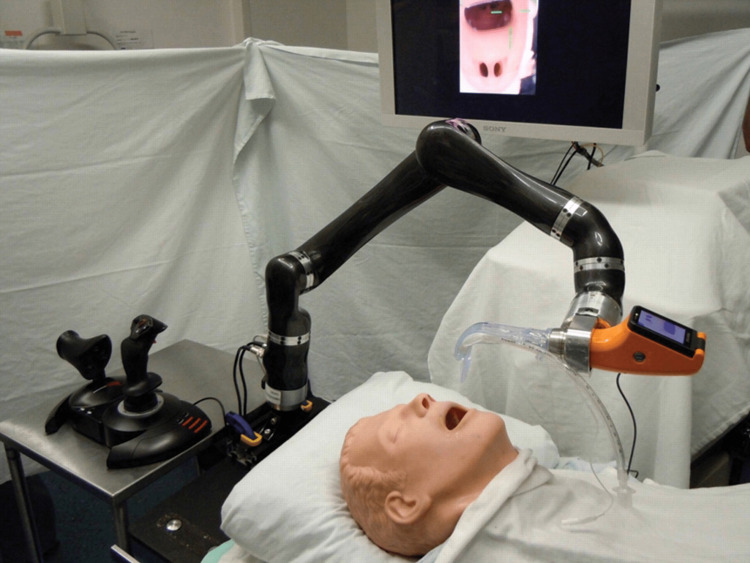
Lateral view of the Kepler Intubation System. Reproduced with permission from Hemmerling et al. [9]. Copyright 2012, Elsevier.

Hemmerling et al. in 2012 designed and developed the Kepler Intubation System (KIS), a dedicated robotic system for endotracheal intubation, and successfully tested it in airway mannequins, including semi-automated operation [8]. The system was named after Johannes Kepler, the renowned German mathematician and astronomer, recognized for his groundbreaking laws of planetary motion. This system comprised a remote control center (joystick and intubation cockpit) linked to a standard video laryngoscope via a robotic arm (Figure 1). In this study, a single operator conducted 90 intubations on an airway trainer mannequin. The intubations were divided into the following three groups: direct view (30 intubations), indirect view where the operator was unable to see the mannequin (30 intubations), and semi-automated (30 intubations). All intubations were successful on the first attempt. The mean intubation times were 46 seconds for the direct view group, 51 seconds for the indirect view group, and 41 seconds for the semi-automated group.

First Tracheal Intubation in Humans With the Kepler Intubation System: A Milestone in Robotic Intervention

**Figure 2:**
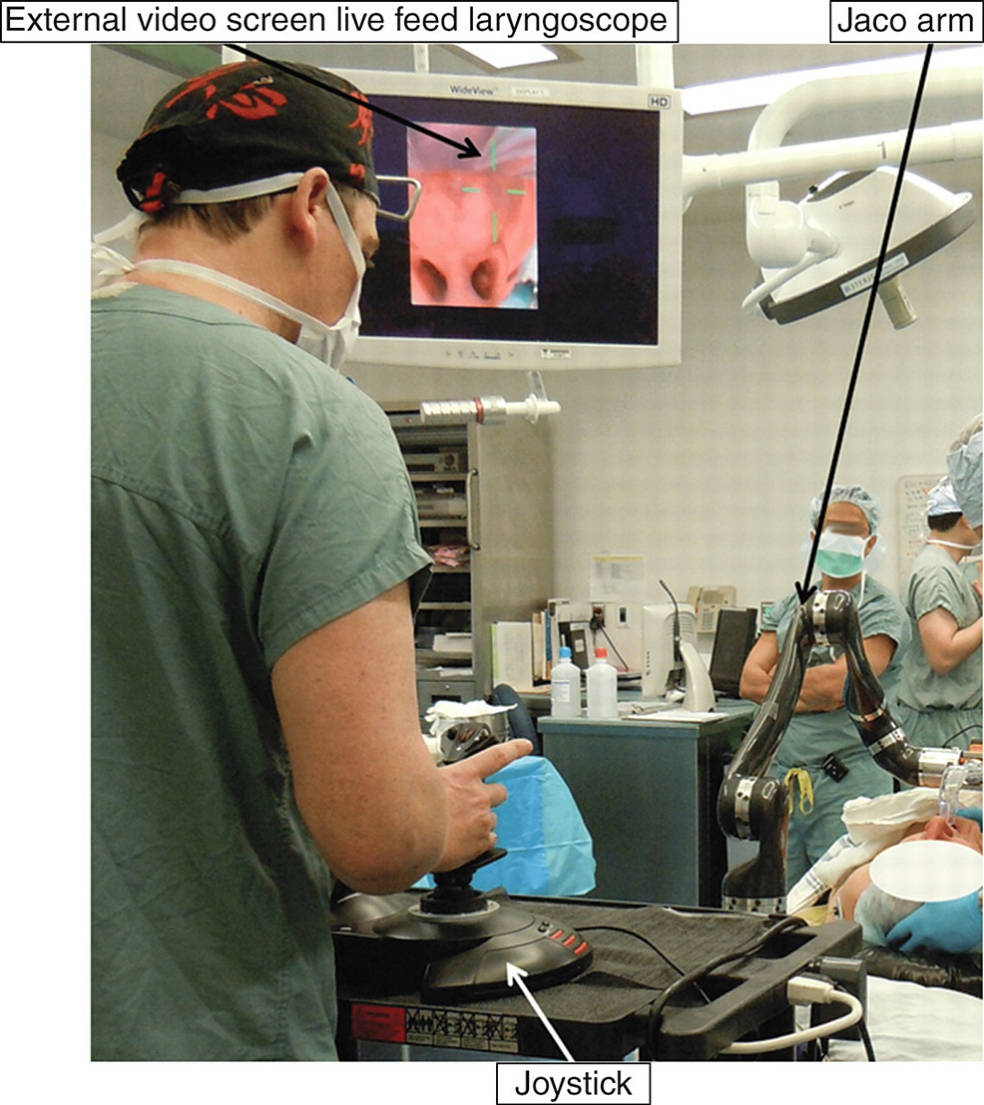
The Kepler Intubation System used for endotracheal intubation in humans. Reproduced with permission from Hemmerling et al. [9]. Copyright 2012, Elsevier.

Hemmerling and his colleagues followed up with the first human studies using the KIS [9]. They conducted a pilot study with 12 patients (11 men and one woman) requiring tracheal intubation for elective surgical procedures. The primary objective was to evaluate the procedural success, intubation time, and complications. Tracheal intubation was successful at a rate of 91.7% (11 out of 12 patients), and the mean time for intubation was 93 seconds with no complications observed. The presence of fogging hindered the successful utilization of the KIS in one patient. The high success rate and reasonable intubation time reported in this study indicated the potential of the Kepler system to enhance procedural efficiency and patient care. The use of real-time video guidance and the precise control offered by the robotic arm may have contributed to improved accuracy in tube placement, minimizing the risk of complications such as esophageal intubation or vocal cord trauma (Figure 2).

However, this study had some limitations. The small sample size and non-randomized design restricted the generalizability of the findings. Further studies with larger sample sizes and randomized controlled trials are warranted to confirm the safety and efficacy of the Kepler system and compare it to conventional intubation techniques. Moreover, the KIS is currently a prototype, and future efforts can be directed toward enhancing its technical aspects by making it smaller, portable, and more semi-automatic.

The successful implementation of robotic tracheal intubation described in this study also raises questions regarding the potential impact on the training and skill acquisition of healthcare professionals. The learning curve associated with robotic technology should be further explored, along with the cost-effectiveness of implementing such systems in clinical practice.

Overall, the study by Hemmerling et al. highlights the promising role of the KIS in orotracheal intubation and encourages further research and development in the field of robotic-assisted airway management.

Remote robot-assisted intubation system

Physicians typically achieve higher success rates in pre-hospital endotracheal intubation compared to paramedics, who generally experience lower success rates [10]. To address this issue, Wang et al. in 2018 developed a remote robot-assisted intubation system (RRAIS) with the expectation of enhancing the success rate of pre-hospital endotracheal intubation [11]. The RRAIS comprised the following four primary components: an intubation robot, a control system, a laptop, and a joystick. Among these components, the intubation robot took center stage as it consisted of essential elements such as a tongue depressor, posture mechanism, and feeding mechanism to push the tube forward. These components collectively formed the crucial backbone of the robot system. To assess the viability of the robot system, a total of 20 pigs were intubated using either a direct laryngoscope or the robot system. Throughout the experiment, intubation time, success rate, and any airway complications were carefully recorded. The robot system demonstrated higher success rates for intubation compared to the laryngoscope, with a first-attempt success rate of 80% versus 40% and an overall success rate of 90% versus 60%. However, when using the mechanical robot, the total intubation time was found to be longer compared to the group using direct laryngoscopy (75 seconds vs. 53 seconds; p-value <0.01). The authors concluded that developing a remote robot-assisted intubation system was highly promising. It held the potential to significantly enhance the success rate of pre-hospital endotracheal intubation and revolutionize the existing rescue model.

The remote control capability enables expert guidance in challenging or remote settings, expanding access to specialized care. The precise control and enhanced visualization offered by the robotic system can potentially reduce complications associated with traditional intubation methods. However, it is worth mentioning that the study primarily concentrated on demonstrating the technical feasibility and proof of concept, with limited clinical data. To fully evaluate the effectiveness, safety, and long-term outcomes of the system, further research involving larger-scale studies and clinical trials is necessary.

IntuBot: designing and prototyping a robotic device for intubation

Cheng et al. in 2018 developed a robotic prototype called IntuBot aiming to automate the intubation procedure. The hardware system included a stepper motor for steering the stylet and two servo motors for generating bending motions at the stylet tip. Additionally, a real-time, vision-based navigation algorithm guided the stylet to locate the vocal cords and target the destination. To conduct pre-clinical testing, they created a silicone model of the airway using three-dimensional printing and CT scan images. The prototype was also evaluated for its steering capabilities [12]. It remains to be seen if IntuBot becomes more widely available and used.

Robotic Endoscope Automated Via Laryngeal Imaging for Tracheal Intubation

**Figure 3:**
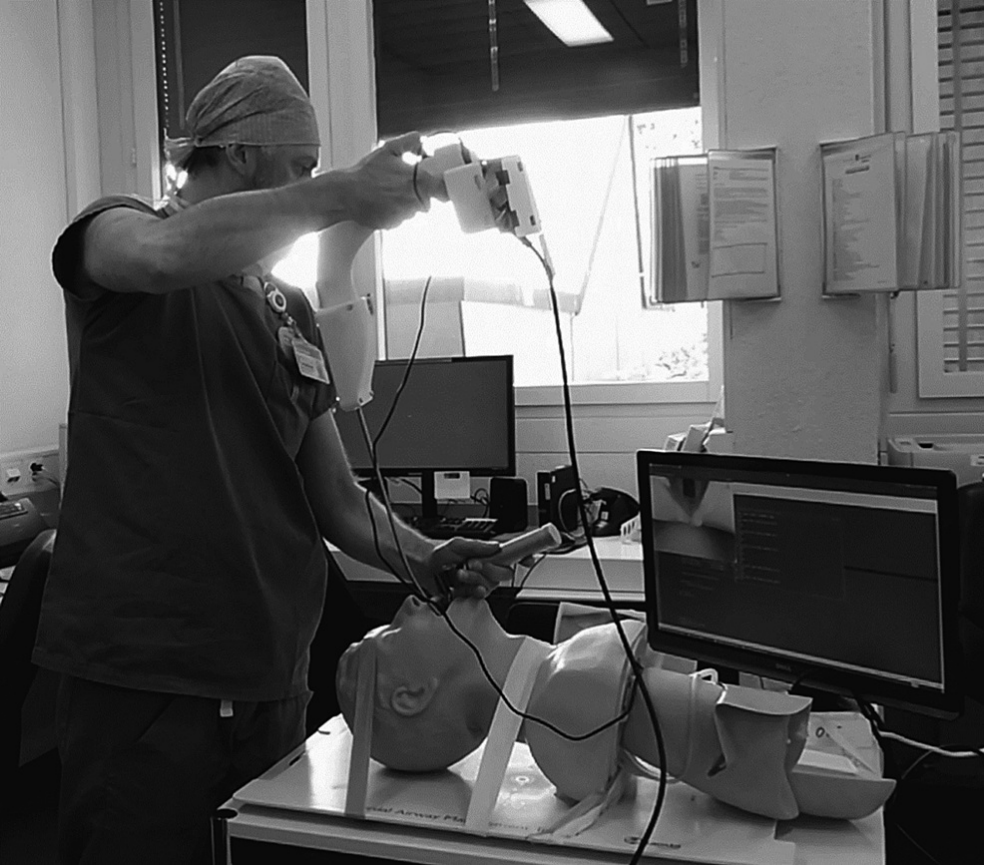
The REALITI system setup. Reproduced with permission from Biro et al. [13]. Copyright 2020, John Wiley and Sons.

**Figure 4:**
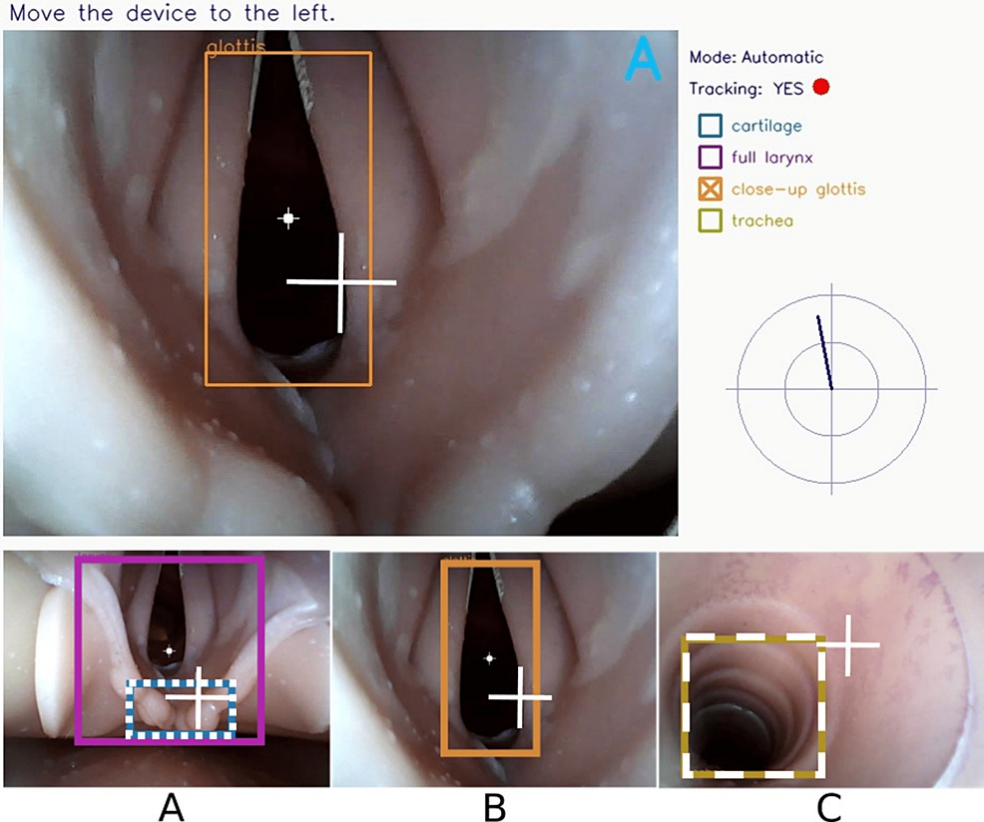
The REALITI system. The user interface comprises various components: (a) the live video feed from the tip camera, (b) feedback on the device configuration, and (c) the detection of anatomical features. The successful recognition of the laryngeal inlet is indicated by a square, while the detected entrance of the glottis is represented by a white dot. The white cross indicates the direction of the tip (A, B, and C). Reproduced with permission from Biro et al. [13]. Copyright 2020, John Wiley and Sons.

In another significant development in the field of robotic intubation, Biro et al. conducted a proof-of-concept study using a manikin to explore the potential of the Robotic Endoscope Automated Via Laryngeal Imaging for Tracheal Intubation (REALITI) system [13]. It consisted of a video endoscope that enables manual control of the endoscopic tip via a joystick, similar to a flexible bronchoscope (Figure 3). It also incorporated image-recognition software that enables automated movements of the tip toward the glottis when specific anatomical features are detected (Figure 4). Once the automated mode is engaged, the tip moves toward the geometric center of the glottis.

The study found that using the automated mode resulted in faster tracheal access compared to the manual mode. Additionally, with increasing user experience, the desired endpoint was reached more rapidly. These findings align with the goals of the REALITI project, which aims to enhance successful tracheal intubations for both anesthetists and non-anesthetists.

Nevertheless, this study has some limitations that need to be acknowledged. First, the actual performance of tracheal intubation was not evaluated, which is a crucial endpoint in assessing the clinical efficacy of such devices. Second, the study did not compare the REALITI device with traditional methods of tracheal intubation such as direct laryngoscopy or video laryngoscopy. Lastly, the observed speed difference of 5.5 seconds between manual and automated modes lacks clinical relevance. These limitations highlight areas for improvement in future studies. Robotic models that have been tested for endotracheal intubation are summarized in Table 2.

**Table 2:** Summary of robotic models tested for endotracheal intubation.

Authors/Year	Robotic model	Subjects	Comparator	Key findings
Tighe et al. (2010) [7]	DaVinci Surgical System Type S (DVS)	Airway simulation mannequin	None	Successful intubation in oral and nasal approaches with 75 seconds and 67 seconds, respectively
Hemmerling et al. (2012) [8]	The Kepler Intubation System (KIS)	Airway trainer mannequin	None	Successful intubation. Mean intubation times: direct view – 46 seconds, indirect view – 51 seconds, semi-automated – 41 seconds
Hemmerling et al. (2012) [9]	The Kepler Intubation System (KIS)	Twelve patients ( 11 male, 1 female)	None	91.7% success rate in tracheal intubation within 100 seconds (11/12 patients)
Wang et al. (2018) [11]	Remote robot-assisted intubation system (RRAIS)	Animals (20 pigs)	Direct laryngoscopy vs. robotic intubation	Mechanical robot had higher success rates (90% vs. 60%) but longer intubation time (75 seconds vs. 53 seconds) compared to direct laryngoscopy
Biro et al. (2020) [13]	Robotic Endoscope Automated Via Laryngeal Imaging for Tracheal Intubation (REALITI)	Airway mannequin	Manual vs. automated mode of REALITI device	Automated mode provided faster tracheal access than manual mode

Advancements in artificial intelligence for airway management: transforming patient care

AI or machine learning algorithm has been employed to predict difficult airway, difficult laryngoscopy, and difficult intubation by incorporating various predictors (physical and radiological). This predictive tool has the potential to accurately anticipate difficult airway cases. Brown et al. introduced an AI system that employs a “semantically embedded neural network” to detect the endotracheal tube (ETT), trachea, and carina. The system provides decision support through ETT detection assistance and position check alerts, aiming to aid intensive care unit physicians at the point of care. By utilizing spatial relationships within the semantic network, the system performs hierarchical segmentation, with tracheal segmentation guiding the detection of the carina and ETT using convolutional neural networks. Spatial relationships further define the ETT tip and safe zone regions [14].

Hayasaka et al. developed an AI model for tracheal intubation by inexperienced medical staff. Facial images were classified based on difficulty, and a deep learning-based convolutional neural network created an AI classification model. The model accurately identified expected intubation difficulties with 80.5% accuracy, 81.8% sensitivity, 83.3% specificity, and an area under the curve of 0.864 (95% CI = 0.731-0.969) [15]. Machine learning has been utilized in various studies to enhance physical examination by automatically analyzing facial features and detecting indicators of difficult airways [16,17]. It has also proved valuable in monitoring pediatric airways, aiding in the early detection of critical incidents (such as acute airway obstruction, esophageal intubation, and hypoxia), and providing timely alerts to clinicians [18]. Moreover, AI and machine learning are being tested to distinguish normal, obstructive, and interstitial lung disease.

Furthermore, advanced robotic technology is increasingly used in the medical field for developing active training systems. The Waseda Kyotokagaku Airway series meets the criteria of providing quantitative information, simulating real-world conditions, and ensuring training effectiveness [19].

Robotic innovations in airway management for COVID-19: enhancing safety and efficiency

The application of teleguidance in medicine is not a new concept [20,21]. During the COVID-19 pandemic, it was reutilized as a strategy to minimize the exposure of staff performing intubation. Robotic assistance has the potential to greatly enhance the safety and efficiency of airway management in infectious conditions, addressing the challenges associated with intubation procedures. A scoping review examining the use of teleguided technology for tracheal intubation revealed that it facilitated intubation effectively, comparable to in-person supervision, without additional complications. Furthermore, it promoted gradual independence for trainees in airway management [22]. However, it is essential to conduct clinical studies to thoroughly assess both the benefits and limitations of this technology. Another unique application of robots is in tracheostomy. Xiao et al. (2020) designed a robotic prototype known as the Robotic-Assisted Trans-Oral Tracheostomy System that drills a hole from within the trachea and outwards. Operators can see the drilling position using a camera for percutaneous tracheostomy [23].

Future directions

Automation and Artificial Intelligence

The integration of automation and AI has the capacity to bring about a transformative revolution in the field of robotic endotracheal intubation. By integrating AI algorithms and machine learning techniques, robotic systems can analyze real-time data, interpret anatomical features, and optimize tube placement automatically.

AI algorithms can assist in identifying key landmarks, guiding robotic movements, and providing real-time feedback to healthcare professionals. This can significantly enhance the precision and efficiency of the intubation process. Additionally, AI algorithms can analyze patient data to predict difficult airways, enabling anesthesiologists to plan and prepare proactively. AI can aid in the detection and prevention of complications, such as accidental esophageal intubation or tube misplacement.

Telementoring and Remote Guidance

Robotic systems can facilitate telementoring and remote guidance, enabling experienced clinicians to provide real-time assistance and guidance during intubations. This capability can enhance training and increase access to expertise in challenging or remote settings [20,21]. Through telecommunication technologies and real-time video conferencing, experts can observe the procedure and provide real-time feedback, enhancing the safety and success rates of robotic intubations. This approach holds particular promise for underserved areas or remote locations where access to specialized healthcare professionals may be limited.

Multimodal Imaging and Navigation

Combining robotic systems with multimodal imaging and navigation technologies can enhance accuracy and visualization during endotracheal intubation. Integration with advanced imaging modalities, such as ultrasound or augmented reality, can provide real-time anatomical information, enabling clinicians to navigate the airway with greater confidence.

Furthermore, robotic systems can be equipped with navigation features, including electromagnetic tracking or computer-assisted navigation, to guide the placement of the ETT accurately. These multimodal approaches can enhance the safety and efficiency of the procedure, particularly in complex or challenging cases.

Ethical and Legal Considerations

The adoption of robotic technology in healthcare raises ethical and legal considerations. Patient safety, privacy, informed consent, and liability are crucial aspects that need to be carefully addressed when implementing robotic endotracheal intubation [24]. It is crucial to establish well-defined protocols, guidelines, and regulations to effectively address ethical and legal considerations, as well as safeguard the rights of patients.

Challenges and limitations

Despite the progress made in robotic intubation, several challenges and limitations remain. Currently, none of these airway robots have been implemented in routine clinical practice. The high cost of robotic systems poses a barrier to widespread adoption, as hospitals and healthcare facilities need to evaluate the financial implications.

Future studies should focus on the feasibility and effectiveness of robotic intubation in challenging scenarios, such as in patients with limited mouth opening, obese patients, and cervical spine immobilization, where precise and controlled placement of the ETT is essential. Additionally, specialized training and expertise are required to operate robotic systems effectively. Anesthesiologists must receive adequate training to ensure proficiency in robotic intubation techniques. The current robotic system for endotracheal intubation has several limitations. One limitation is intubation time which may lead to airway trauma and prolonged sympathetic stimulations. These models need to be fine-tuned further to mitigate these complications. Furthermore, technical difficulties, such as robotic system malfunctions or errors, may arise during the procedure. Addressing cost concerns, providing comprehensive training programs, refining technical capabilities, and establishing ethical frameworks can contribute to overcoming these challenges and maximizing the potential benefits of robotic endotracheal intubation. As robotic endotracheal intubation continues to evolve, the field will benefit from robust clinical trials, comparative studies, and outcomes.

This review incorporates airway-related robotics and AI to emphasize the significance and need for advancements in this field. However, it does not cover all aspects of robotic intubation in one paper, nor does it encompass all evolving aspects. The limitations of this paper lie in its focus solely on intubation-related robotics and AI, omitting other developments in airway management. Additionally, due to constraints, a comprehensive review of practicalities, global availability, usage, and pros and cons could not be presented.

## Conclusions

Although AI and robotics in airway management show potential, uncertainty remains. AI in airway management is in its early stages, and further research is vital to grasp its potential impact on patient outcomes. Robotic intubation improves precision, but data limitations hinder definitive conclusions. AI should complement the expertise of anesthesiologists. AI-guided or robotic-assisted airway management requires further high-quality studies in diverse patient populations. These innovative solutions may transform service provision and education in the future. Despite their limitations, we should embrace these advancements as opportunities to modernize and progress in the field of airway management. This review lays the groundwork for future research and AI applications.
